# Regional Disparities in Mortality after Ischemic Heart Disease in a Brazilian State from 2006 to 2010

**DOI:** 10.1371/journal.pone.0059363

**Published:** 2013-03-19

**Authors:** Luciano de Andrade, Vanessa Zanini, Adelia Portero Batilana, Elias Cesar Araujo de Carvalho, Ricardo Pietrobon, Oscar Kenji Nihei, Maria Dalva de Barros Carvalho

**Affiliations:** 1 Department of Nursing, State University of the West of Parana, Foz do Iguaçu, Parana, Brazil; 2 Research on Research Group, Duke University Health System, Durham, North Carolina, United States of America; 3 Department of Surgery, Duke University Health System, Durham, North Carolina, United States of America; 4 Department of Medicine, State University of Maringa, Maringa, Parana, Brazil; Universidade Federal do Acre (Federal University of Acre), Brazil

## Abstract

**Background:**

High technology in the field of interventional cardiology applied in tertiary hospitals has brought enormous benefits in the treatment of ischemic heart disease (IHD). However, IHD mortality rates remain high. We analyzed the relationship between IHD mortality rate and the socioeconomic, demographic, and geographic conditions in 399 cities in Parana state, Brazil, from 2006 to 2010.

**Methods and Results:**

Data were obtained from the Mortality Information System and the Brazilian Institute of Geography and Statistics and evaluated through Exploratory Spatial Data Analysis. GeoDa™ was used to analyze 29.351 deaths across 399 cities. We found a positive spatial autocorrelation regarding IHD mortality (I = 0.5913, p = 0.001). There was a significant positive association between each of three socioeconomic and demographic indicators and IHD mortality rate: Population Elderly Index (I = 0.3436), Illiteracy Rate (I = 0.1873) and City Development Index (I = 0.0900). In addition, two indicators presented significant negative association with IHD mortality rate: Adjusted Population Size (I = −0.1216) and Gross Domestic Product (I = −0.0864). We also found a positive association between IHD mortality rates and the geographic distances between patients’ city of residence and their corresponding regional referral centers in interventional cardiology (I = 0.3368). Cities located within Regional Health Units with Reference Interventional Cardiology Center presented a significantly lower average specific mortality rate by IHD. The high mortality rate by IHD within the Regional Health Units was not restricted to socioeconomic and demographic variables, but dependent on the distance between each city and their reference interventional cardiology center.

**Conclusions:**

We conclude that geographic factors play a significant role in IHD mortality within cities. These findings have important policy implications regarding the geographic distribution of cardiac health care networks in Latin America and in other emerging countries.

## Introduction

Ischemic Heart Disease (IHD) causes 12.8% of all deaths in both developed and developing countries, making it the most prevalent cause of death in these locations [1]. This high mortality percentage is mainly caused by pre-hospital delays in the treatment of patients living in areas without specialized services or nearby interventional cardiology units [2]–[5]. Although Brazil does not have exact estimates of individuals seeking health services after symptoms suggestive of IHD, according to a national healthcare database in 2010 there were 221,898 hospital admissions for IHD, with 99,725 deaths (8.77% of the overall total of deaths in the country), with 37,688 people dying before reaching a hospital [6]. In developing countries, patients with IHD rarely receive immediate treatment, and as the prevalence of this disease increases, the mortality rate due to IHD is likely to proportionally increase [7].

In Brazil, since 1988 an Unified Health System (SUS) has been implemented with the aim of providing a universal and equitable access to all levels of health care services to the population [8]. Although different health care indicators have clearly improved since its implementation, an example being an overall decrease in mortality secondary to cardiovascular diseases, several of its organizational features have imposed barriers in delivering adequate healthcare to patients with ischemic heart diseases [8], [9]. Some of these current characteristics include a focus on primary and acute care rather than prevention and education, the problematic provision of secondary care, poor supply of cardiologists and diagnostic examinations, underfunded public service system, and socioeconomic inequity between the coverage of public and private healthcare systems [8], [9].

We currently know that lower socioeconomic and demographic conditions lead to higher IHD death rates [10]–[16], as well as that long distances from the IHD event and the reference cardiology center is a risk factor for IHD [3]. Factors contributing to morbidity and mortality secondary to IHD have been a topic of constant debate. Patients who are poor, with low educational level, or residing in geographical locations distant from large urban centers all have problems accessing health care services [3]–[5], [17]–[21]. It has also been demonstrated that deprived neighborhoods present higher rates of mortality due to IHD [22]–[25]. Studies have investigated whether making reference cardiology services more accessible would ultimately reduce mortality rates [26]–[29]. Despite the extensive information about isolated factors, to our knowledge the interplay among distance from a reference cardiology center, socioeconomic/demographic conditions, and IHD-specific mortality rate have not been evaluated in developing countries.

Analyzing the connection among these factors was therefore the main aim of our study, specifically focusing on the socioeconomic, demographic and geographic causes of IHD mortality in 399 cities in Parana state, Brazil.

## Methods

### Ethics

Our study was approved by the Institutional Review Board (Comitê de Ética em Pesquisa Envolvendo Seres Humanos) of the State University of the West of Parana (UNIOESTE), Brazil.

### Study Design and Sample Site

This is an observational, cross-sectional, ecological study using spatial analysis techniques based on mortality data from 2006 to 2010 in Parana state, Brazil.

**Figure 1:**
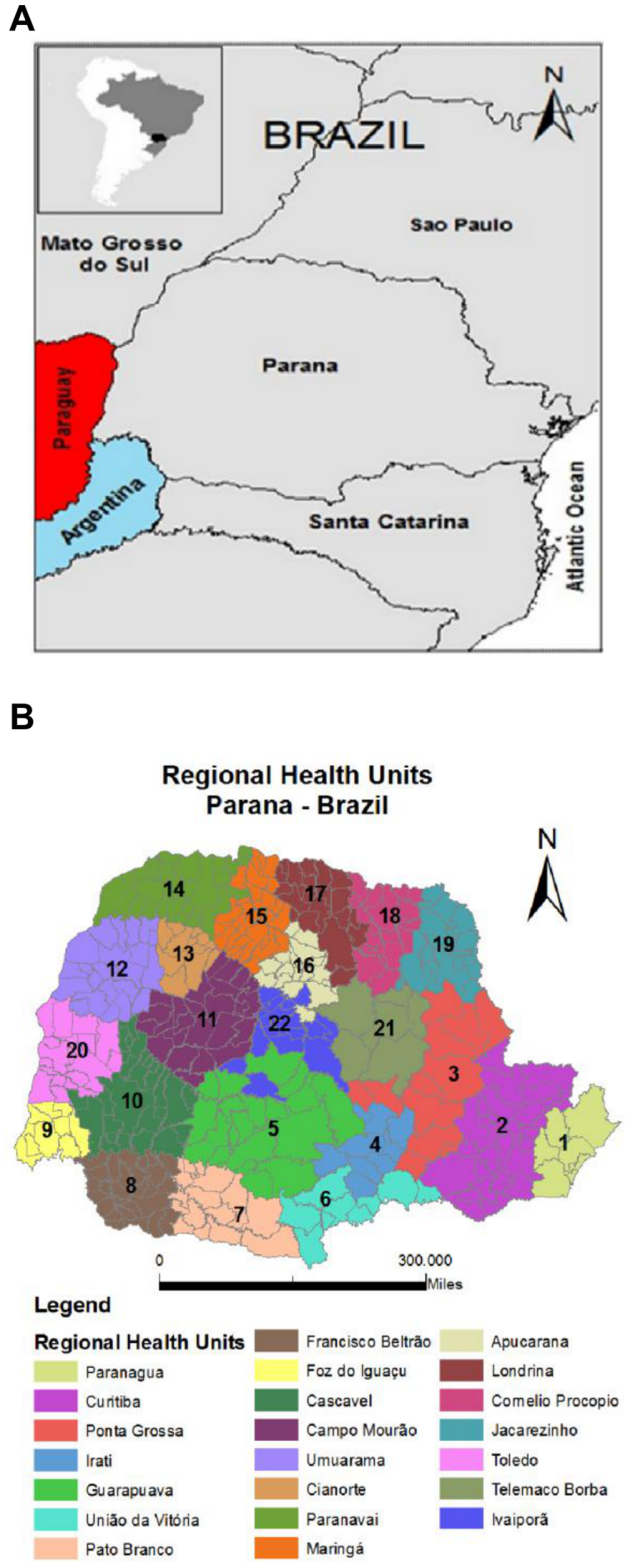
Maps. A) Map of the Parana state. Source: Geographic Atlas of the Parana state, 2011 [30]. B): Regional Health Units in Parana state. Source: Secretary of Health of Parana state (BR), 2012 [34].

Parana state is located in Southern Brazil, occupying an area of 199,880 km^2^, with latitude coordinates 22°30′58′′ and 26°43′00′′ and longitude coordinates 48°05′37′′ and 54°37′08′′ (Figure 1A) [30]. According to the 2010 Brazilian Census, Parana state presented 10,439,601 inhabitants, most of them (85.3%) living in urban area, and making it the 6^th^ most populated in Brazil (5.47% of the total population) [31]. The Elderly Index (population above 65 years old) of Parana state was 7.6% in 2009, and therefore similar to the Brazilian average of 7.4% [31], [32]. Parana state presented a Gross Domestic Product (GDP) occupying the 5^th^ rank in the country, representing 5.76% in 2010 [32] and a Human Development Index (HDI) of 0.846 (6^th^ of the country). This value is above the Brazilian average HDI of 0.816 [33].

Parana state has 399 cities administratively grouped into 22 Regional Health Units responsible for health care management (Figure 1B) [34].

### Data Sources

We obtained data from the Mortality Information System of the Ministry of Health (SIM/MS) [35] and from the Brazilian Institute of Geography and Statistics (IBGE) [31]. We used population data from the IBGE to calculate the population per city. It was calculated by considering the total number of citizens over age 20 in each city throughout the period and dividing the result by the period of study in years. Hence, we obtained the average number of inhabitants per city for the years 2006 to 2010. The social and economic data of the period of 2009–2010 regarding cities were available online at IBGE and the Parana Institute of Economic and Social Development (IPARDES) [31], [32].

The map with the geo-referenced base of Parana state, entitled Political-Administrative Division of Parana State in the Year 2010, was made available for free on the Internet in shapefile (SHP) and online through of the Institute of Cartography and Land Geosciences [36].

To assemble a death profile related to IHD, we evaluated socioeconomic and demographic factors according to patientś city of residence. We analyzed five socioeconomic and demographic indicators for each city: Population Elderly Index (ratio between the population over 65 years old and the population under 15 years of age) [31]; Illiteracy Rate (percentage of illiterate people 15 years of age and older with less than three years of formal education) [31]; Gross Domestic Product per capita [31], [32]; City Development Index (measures the performance of management and public actions of the city, considering three areas: employment and income, health, and education) [32]; Adjusted Population Size (city number of individuals over 20 years old - average considering the period of 2006 to 2010) [31]. We selected IHD cases using the International Statistical Classification of Diseases and Related Health Problems –10^th^ Revision (ICD-10) [37], specifically as codes I20 to I25. We used an empirical Bayes spatial estimator to minimize variance in mortality rates by city, due to the variability associated with rates expressing the likelihood of a given event when the rate and population are both small. In other words, this combination might cause random fluctuations. The global empirical Bayes estimator calculates a weighted average of the gross rate of the locality and the region’s global rate (ratio between the total number of cases and the total population) [38]. Specific mortality rates per 100,000 inhabitants were obtained for each of the 399 cities in Parana state.

### Spatial Analysis

We analyzed spatial data grouped by geographic areas (polygons), to evaluate whether the presence of spatial aggregation was associated with socioeconomic, demographic, and/or geographic variables [39]–[40]. We used Exploratory Spatial Data Analysis (ESDA) and the software package GeoDa™ version 0.9.5-i (Spatial Analysis Laboratory, University of Illinois, Urbana-Champaign, IL, USA) to determine measures of global spatial autocorrelation and local spatial autocorrelation [41]. To evaluate the existence of spatial autocorrelation, we defined a spatial weight matrix - *W.* This matrix allows for the measurement of non-random association between the value of a variable observed in a given geographical unit with the value of variables observed in neighboring units. Furthermore, we used the binary matrix-type Queen, which attributes a value of one for neighbors in any spatial location within the analyzed region [42].

We calculated spatial autocorrelation evaluating mortality rates, socioeconomic, and demographic indicators for each city using the (I) Global Moran index for univariate and bivariate analysis [42], [43]. This index measures both the spatial autocorrelation and the weighted neighborhood matrix, indicating that the IHD mortality rates of a given region might be similar to those of neighboring regions. Values of Moran’s I vary between −1 and +1. Values greater or smaller than the expected value of Moran’s I [E (I) = −1/(N - 1)] indicate a positive or negative autocorrelation, respectively. If the value of Morańs I is 0 (zero), the region is considered to have spatial independence [42], [43].

Morańs I values between 0 and +1 indicate positive spatial association (direct). This indicates that regions with high Morańs I values for the variable in question are surrounded by regions which also have high variable values (high/high). Similarly, regions with low variable values are surrounded by neighbors who also have low variable values (low/low). Negative values of Morańs I (from 0 to −1) represent negative spatial association (reverse). Therefore, regions with high Morańs I values are surrounded by regions with low variable values, while regions with low Morańs I variable values are surrounded by neighbors with high variable values [39], [42], [43].

A limitation of Global Morańs I is that it can hide local patterns of spatial association since negative values of Morańs I do not necessarily indicate the absence of spatial correlation at the local level [43]. To identify patterns of spatial association that were significant and specific to each analyzed area, we used local indicators of spatial association (LISA). LISA allowed us to identify the existence of spatial clusters, or sites with high or low values for the analyzed variables, ultimately determining regions that can contribute to spatial autocorrelation [42]. Choropleth maps were generated to investigate the presence of mortality rate clusters. These values were divided by class intervals and aggregated into tracks of standard deviation in relation to average. Coefficients of global and local spatial autocorrelation were considered significant at p<0.05. These coefficients were analyzed by pseudo significance levels. In other words, they were confirmed through the redistribution of simulated values and examined areas using permutation tests [44].

We used Google Maps [45] featuring satellite images to determine distances between cities and the Reference Interventional Cardiology Centers. We used the Mann Whitney test for independent samples to compare the specific mortality rates of cities belonging to Regional Health Units with reference services to cities with Regional Health Units that lack reference services. For data processing, we used GraphPad Prism v. 5.0. (GraphPad Software, Inc., San Diego, CA, USA).

## Results

During the period covered by our study there were 29,351 deaths due to IHD, mainly among men (60.30%), between 60 and 79 years of age (53.07%), white (85.74%), married (53.55%), and with an educational level of up to three years in school (58.29%). Most deaths occurred in the morning (28.79%), followed by the afternoon (26.08%).

**Figure 2:**
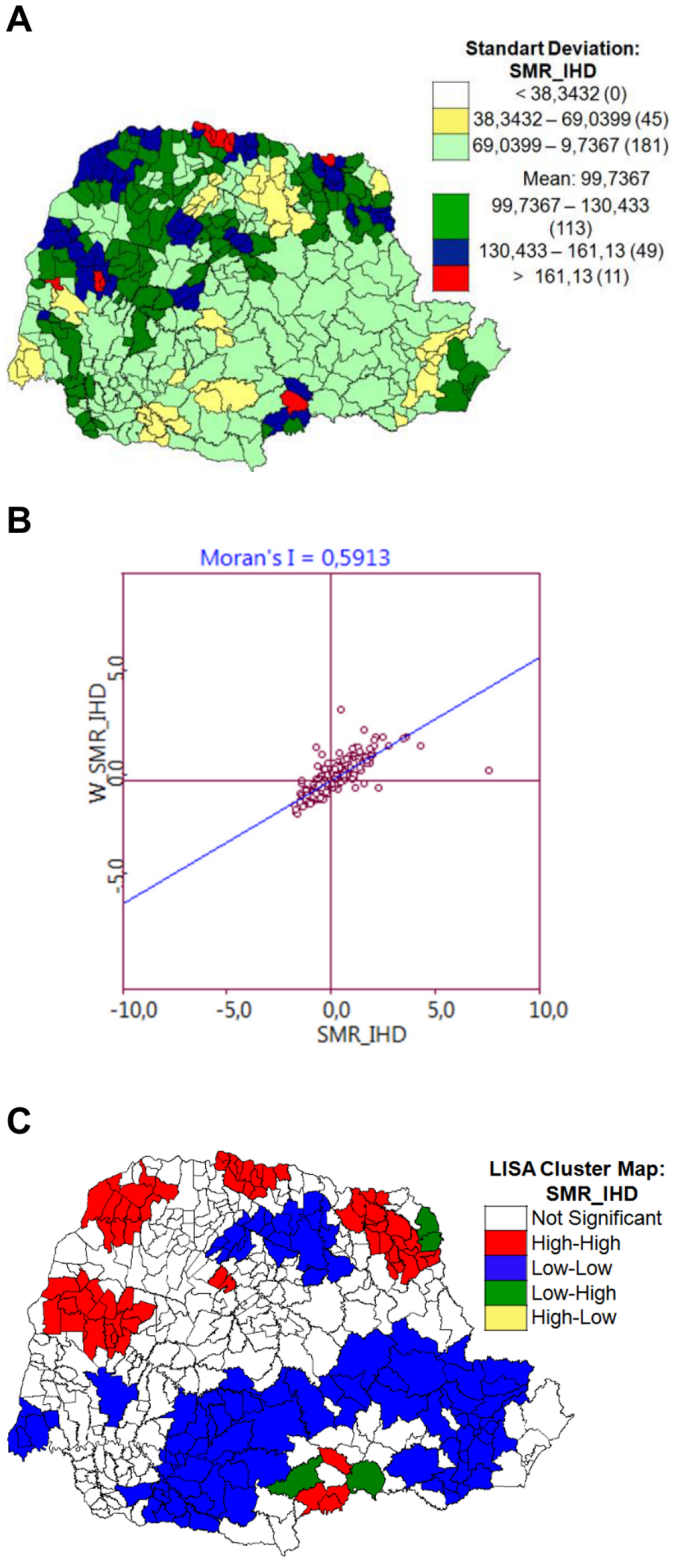
Exploratory spatial analysis of specific mortality rate by IHD in state of Parana, Brazil, 2006–2010. A) Spatial distribution of cities’ specific mortality rate (SMR) by IHD, with ranges of standard deviation from the average for the delimitation of class intervals; the number of cities is in parenthesis. B) Moran’s diagram of dispersion (univariate analysis) of specific mortality rate (SMR) by IHD (X axis: city’s SMR; Y axis: weighted average SMR of the neighbor cities). C) LISA univariate analysis: cluster formation according to specific mortality rate (SMR) by IHD (Types of cluster: high-high; low-low; low-high, high-low).

Regarding spatial patterns of death distribution from IHD in the 399 cities in Parana state, on average 99.74/100,000 inhabitants over the age of 20 died from IHD. Out of 399 cities, most cities (45.40%) presented IHD mortality rates between 69.04 and 99.74 per 100,000 inhabitants, these cities being located mainly in east, southeast, central south and south-west regions of the Parana state. Rates above 134.43/100,000 inhabitants were identified in 60 cities located mainly in northeast, north, central north, and northwest regions (Figure 2A).

Univariate analysis (Figure 2B) regarding specific mortality rates by IHD indicated the existence of a positive spatial autocorrelation (I = 0.5913, p = 0.001), demonstrating that cities with high mortality rates by IHD tend to be surrounded by neighboring towns with similar values, so also with high mortality rates by IHD. LISA analysis allowed the detection of clusters based on similarities between cities (Figure 2C). We could therefore classify these groups of cities using the following categories: high-high, i.e., cities with high rates of death from IHD with surrounding neighbors also displaying high rates of death from IHD; low-low, i.e., cities with low death rates from IHD with neighbors with low IHD mortality rates; and low-high, i.e., cities with low death rates from IHD with neighbors with high IHD mortality rates. Our analysis did not demonstrate any significant high-low type of cluster formation, i.e., cities with high rates of death from IHD with neighbors with low IHD mortality rates (Figure 2C).

We identified six high-high type of clusters which involved cities located in the following Regional Health Units: **1)** 10^th^ (four cities), 11^th^ (two cities), 12^th^ (eight cities), 20^th^ (three cities); **2)** 12^th^ (three cities), 14^th^ (eight cities); **3)** 14^th^ (two cities ), 15^th^ (five cities), 17^th^ (five cities)**; 4)** 18^th^ (seven cities), 19^th^ (eleven cities); **5)** 11^th^ (two cities)**; 6)** 6^th^ (three cities).

**Figure 3:**
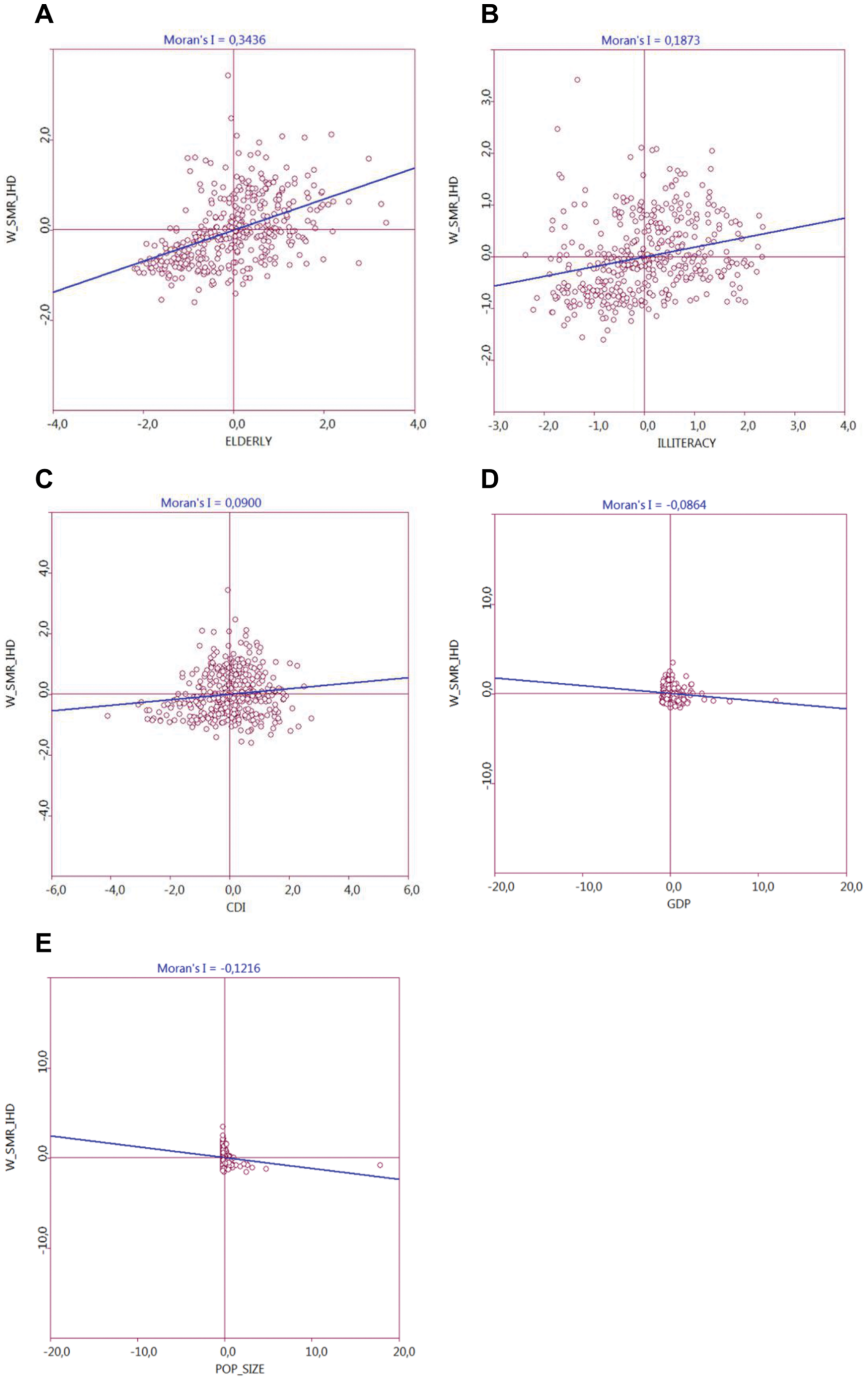
Moran’s diagram of dispersion (bivariate analysis). Analysis of socioeconomic or demographics variables of the city of residence of the patient (X axis) and the weighted average specific mortality rate by IHD of the neighbor cities (Y axis). A) Population Elderly Index. B) Illiteracy Rate. C) City Development Index (CDI). D) Gross Domestic Product (GDP). E) Adjusted Population Size.

Figure 3 demonstrates that all five socioeconomic and demographic indicators used for analysis in this study were significantly associated with the specific mortality rate by IHD (p<0.05). The correlation was positive for the Population Elderly Index (I = 0.3436, P = 0.001), Illiteracy Rate (I = 0.1873, p = 0.001) and City Development Index (I = 0.0900, P = 0.001). The positive correlations indicated that cities with a high level of these indicators were surrounded by cities with high specific mortality rates by IHD. However, specific mortality rates by IHD correlated negatively with Gross Domestic Product (I = - 0.0864, P = 0.001) and Adjusted Population Size (I = - 0.1216, P = 0.001), indicating that cities with high values of Gross Domestic Product and/or Population Size were surrounded by cities with low specific rates of mortality by IHD and vice versa. Stated in a different way, socioeconomic and demographic factors significantly influence the number of deaths by IHD in these cities and thus may be related with the observed citieś clustering pattern.

**Figure 4:**
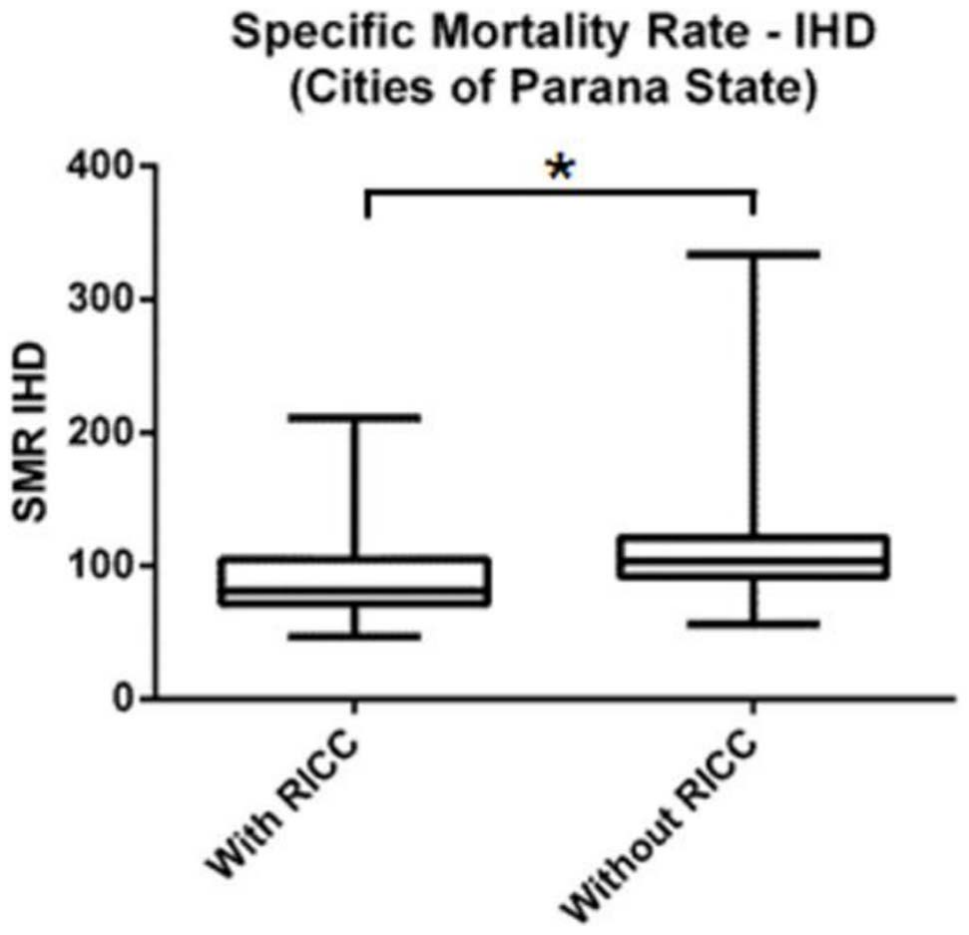
Boxplot comparing specific mortality rate by IHD among cities with and without Reference Interventional Cardiology Centers (RICC). * p<0.001.

In addition to these parameters, we also investigated the possible influence of the Reference Interventional Cardiology Centers within Regional Health Units in the specific mortality rate by IHD in the cities. We compared the specific mortality rate by IHD of cities located within Regional Health Units with Reference Interventional Cardiology Centers (n = 200) with those from the cities located in Regional Health Units without Reference Interventional Cardiology Centers (n = 199). The cities located within Regional Health Units with Reference Interventional Cardiology Centers presented a significantly lower average specific mortality rate by IHD (90.46±29.92 deaths/100.000 inhabitants) in comparison with the group of cities located in Regional Health Units without Reference Interventional Cardiology Centers (109.1±28.59 deaths/100.000 inhabitants) (Figure 4).

**Figure 5:**
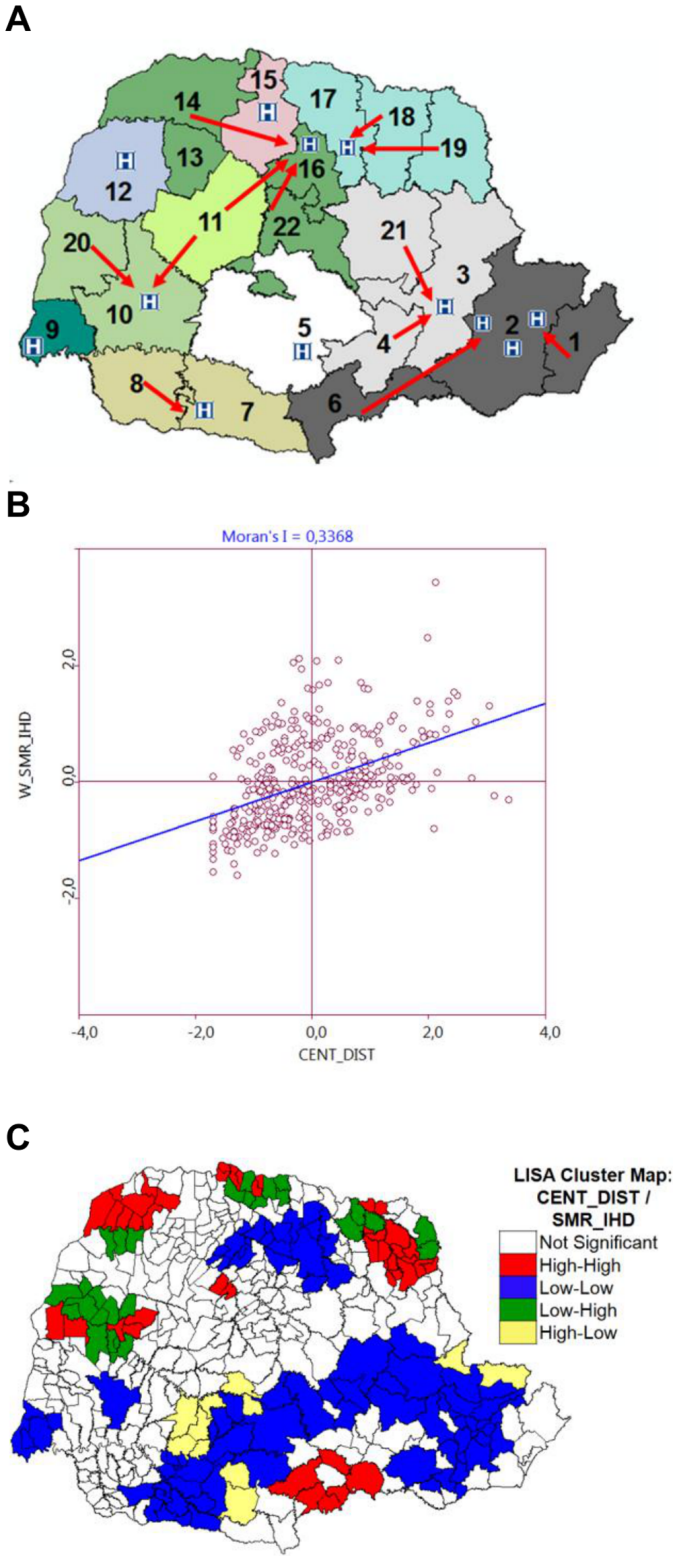
Spatial analysis of distance of the Reference Interventional Cardiology Centers and the mortality by IHD. A) Map with the approximate localization of the services of high complexity in cardiovascular and interventional surgery in Parana state, Brazil (arrows: indicate the localization of the Reference Interventional Cardiology Centers where the neighbor cities direct the IHD patient [Adapted from: Regional Master Plan/SESA, 2009]) [46]. B) Moran’s diagram of dispersion showing the relationship between the distance of the Reference Interventional Cardiology Centers to the patients residence city (X axis) and the weighted average of the specific mortality rate (SMR) by IHD of the neighbor cities (Y axis). C) LISA bivariate analysis: cluster formation according to the distance of the Reference Interventional Cardiology Center to the patients city of residence and the weighted average specific mortality rate (SMR) by IHD data of the respective neighbor cities (cluster type: high-high; low-low; low-high, high-low).

We further analyzed the influence of the existence of Reference Interventional Cardiology Centers on the specific mortality rate by IHD among neighbor cities. Figure 5A presents a map displaying the distribution of Reference Interventional Cardiology Centers in Parana state and their respective area of influence according to the current state’s regional master plan. This plan guides the healthcare spatial regionalization process, aiming at the reduction of social and territorial inequalities as well as the improved access to the population at all socioeconomic levels [46]. The univariate Global Moran analysis indicated that the geographical distances between the cities of residence to the Reference Interventional Cardiology Centers showed strong positive spatial autocorrelation (I = 0.7142; p = 0.001), indicating that the cities that are located far from their Reference Interventional Cardiology Center present neighbor cities that also are located far from their Reference Center. Using the bivariate Global Moran analysis, the geographical distance of the patients city of residence to Reference Interventional Cardiology Center also showed a positive spatial autocorrelation with the neighbor cities specific mortality rate by IHD (I = 0.3368; p = 0.001).

This positive correlation demonstrated that cities far from their respective Reference Interventional Cardiology Center more likely present neighbor cities with higher specific mortality rate by IHD (Figure 5B). The bivariate LISA analysis (Figure 5C) indicated the formation of different types of geographical city clustering according to Reference Interventional Cardiology Center distance and specific mortality rate by IHD. The high-high type of clustering was found mainly involving cities of the 6^th^, 11^th^, 14^th^ and 19^th^ Regional Health Units without Reference Interventional Cardiology Center and the 10^th^ and 15^th^ (north region) Regional Health Units with Reference Interventional Cardiology Center. These data indicated that the cities far from Reference Interventional Cardiology Center were surrounded by cities with high specific mortality rate by IHD. Since our data indicated that these cities are located in high-high type of cluster, these data indicated that the patients that lives in cities far from the Reference Interventional Cardiology Center present higher probability to die due to IHD. The cities within 10^th^ and 15^th^ Regional Health Units presented a high-high profile even presenting a Reference Interventional Cardiology Center, possibly due to the fact that these Regional Health Units present great number of cities (25 and 30, respectively), what may increase the distance of the Regional Health Units peripheral cities to the Reference Interventional Cardiology Center. In contrast, the low-low type of clustering involved cities mainly of the 2^th,^ 3^th^, 5^th,^ 7^th^, 9^th^, 15^th^, 16^th^, 17^th^ Regional Health Units, all with Reference Interventional Cardiology Center, and the 4^th^ Regional Health Unit, without Reference Interventional Cardiology Center.

Since the cities within Regional Health Units with a Reference Interventional Cardiology Center (Group 1) presented a lower average specific mortality rate by IHD when compared with cities of the Group 2 without a Reference Interventional Cardiology Center, we also analyzed the association between socioeconomic and demographic variables of these two groups of cities with their respective mortality rates by IHD (Table 1). This analysis indicated that both the Population Elderly Index and the City Development Index presented a positive association with specific mortality rate by IHD in both groups. However, the cities of Group 1 presented a greater Global Moranś I coefficients in comparison to Group 2. The Illiteracy rate had a positive association in group 1 and a non-significant association in group 2. The Gross Domestic Product and Adjusted Population Size presented significant negative association with specific IHD mortality rate only in the cities of Group 1 but not in group 2. The parameter “Geographical distance to Reference Interventional Cardiology Center” was positively associated with the mortality rates for IHD in both groups. However, the higher Global Morańs I coefficient presented by Group 2 in comparison to Group 1, indicated that when the distance is greater between the cities of residence of the patients that died from IHD and the headquarter city of the Reference Center Interventional Cardiology, higher is the mortality rates for IHD.

**Table 1:** Global Moran’s I coefficient of the IHD specific mortality rate and geographic, demographic and socioeconomic indicators of the cities localized within Regional Health Units with or without Reference Interventional Cardiology Center (RICC).

Indicators	Regional Health Units with a RICC		Regional Health Units without a RICC	
	*I*	*p value*	*I*	*p value*
Population Elderly Index	0.4779	0.001	0.0872	0.022
Illiteracy Rate	0.3439	0.001	−0.0125	0.434
Gross Domestic Product	−0.1000	0.001	−0.0187	0.388
City Development Index	0.0965	0.025	0.0709	0.048
Adjusted Population Size	−0.1196	0.001	−0.0420	0.217
Geographical distance to RICC	0.1944	0.001	0.2939	0.001

Overall, these data indicate a decrease in the impact of socioeconomic and demographic parameters on mortality rates for IHD in group 2 compared with group 1, possibly due to the increased distance between the place of residence of the patient and the respective Reference Interventional Cardiology Center, demonstrating that the absence of these reference centers at a regional level can determine a particular pattern of mortality rates for IHD and greater inequality in access to referral centers for interventional cardiology.

## Discussion

To the best of our knowledge, in developing countries, this is the first study to assess the interaction between distance from a center of reference in cardiology, the socioeconomic and demographic conditions and specific mortality rates by IHD. The present study showed a significant univariate positive spatial association for IHD specific mortality rate in the Parana state, i.e., cities with high mortality rates of IHD were surrounded by cities with high mortality rates of IHD, determining a high-high pattern of clustering. Low-low and high-low types of clustering were also observed. Furthermore, there was an association between different socioeconomic, demographic and geographic indicators and higher IHD specific mortality rate; the Population Elderly Index, distance to a Reference Interventional Cardiology Center, City Development Index, and Illiteracy Rate, all were positively associated. In contrast, the Adjusted Population Size and Gross Domestic Product were negatively associated. These data indicate that geographical distribution of Reference Interventional Cardiology Centers as well as socioeconomic and demographic characteristics of the cities influences specific mortality rate by IHD.

Corroborating our findings, other studies have shown that parameters of low levels of socioeconomic development and unfavorable demographic features increase mortality rates by IHD when combined with increased distance from a treatment center [10], [47]–[51]. These studies highlight the importance of social, environmental and geospatial factors in the mortality rate by IHD, which may interact with other factors such as genetic susceptibility, behavioral variables and other biological attributes.

In our study, the variables related with higher mortality rates by IHD were identified using a bivariate analysis indicated by the Morańs I coefficient. Regression analysis or similar approaches have been performed in other studies evaluating risk factors for IHD in different countries such as Sweden [23], United States of America (USA) [15], [25], [52] and China [16]. A study performed in India has evaluated the risk factors for IHD mortality utilizing hazard regression modelling [14]. In these studies, risk factors identified as negatively associated with IHD mortality rates included education [14], [15], [25], income [15], [23], [25], race (black/hispanic) [15] and urban areas [25] while the variables positively related were sex (male) [15], [16] and age [15], [16].

Data obtained by these studies corroborate our findings. The associations observed in the our work might therefore be explained as follows: 1) Low Gross Domestic Product and Illiteracy may decrease patientś condition to seek adequate treatment and/or decrease the comprehension of their own health condition [14], [53]; 2) High Elderly Index may be associated with improvement in the living conditions of the population. However, the increase in life expectancy leads to increased susceptibility to the development of chronic degenerative diseases, not only determined by aging, but also by environmental factors such as life style, physical inactivity, poor nutrition, housing conditions, labor and difficult access to health service [54]; 3) Low Adjusted Population Size may be related with a city’s lower capacity to improve local healthcare system since the municipalities with small population size may present scarcity of resources, lack of investment in transport, equipment and trained professionals [55]; 4) Greater distance to a Reference Interventional Cardiology Center is related with increased time delay and difficulty to access an adequate treatment to the patient; 5) Most of the municipalities of Parana state presents a medium to high City Development Index [32]. Since our results indicate a positive association between IHD specific mortality rate and city development index, city development levels may be related with an increase in the population’s susceptibility to develop ischemic heart disease. Factors involved could be a more stressful lifestyle, inadequate nutrition, pollution, unemployment and violence, with the absence of a suitably established tertiary cardiovascular health care system [48], [56].

When the regional hospitals with interventional cardiology centers were considered, only 10 of the 22 Regional Health Units have referral centers for interventional cardiology. We found that cities belonging to 12 Regional Health Units without a referral center for interventional cardiology have higher average rates of IHD mortality than cities that belong to Regional Health Units with these centers. These findings can be explained by the long distances that patients had to travel between their city of residence and the Reference Interventional Cardiology Center [18], [49], [57], [58]. In Parana state, cities located within the Regional Health Units with Reference Interventional Cardiology Center present shorter distances to the respective regional referral hospitals (average of 36.99 miles) while cities located within Regional Health Units without Reference Interventional Cardiology Center present higher distances to the respective regional referral hospitals (average of 86.81 miles).

Our results indicate that the high mortality rate from IHD within the Regional Health Units are not restricted to socioeconomic and demographic variables, but dependent on the distance of each city to the Reference Interventional Cardiology Center. The absence of a Reference Interventional Cardiology Center within the Regional Health Unit may therefore be an important independent predictor of IHD mortality, in tandem with the importance of socioeconomic and demographic variables.

Other studies have shown that higher rates of IHD mortality are frequently associated with greater distances from a patient’s place of residence to a major reference interventional cardiology center which may increase the time delay to the specialized treatment initiation or even precluding it [2], [13], [47], [48], [49], [59]. However, one study analyzing the accessibility of cardiac interventional services in Alabama and Mississippi (USA) has demonstrated that the distance factor may not influence IHD mortality rate [52]. To a broader comprehension of these differences it is important to notice that the United States presented on average one Reference Interventional Cardiology Center per 585,135 inhabitants, and Alabama and Mississipi presented one Reference Interventional Cardiology Center per 434,521 and 593,459 inhabitants, respectively [60]. The state of Parana, in Brazil, presents approximately one Reference Interventional Cardiology Center per 855,561 inhabitants, which is comparatively almost half the quantity of Reference Interventional Cardiology Center that Alabama State presents.

Thus, the importance of the distance factor on IHD specific mortality rate may be influenced by the number of Reference Interventional Cardiology Center established regionally, which may also influence the entire health care network organization and the assistance that patients will receive, since both are influenced by the nearby number of available tertiary referral centers. In countries like the United States and Italy, the number of Reference Interventional Cardiology Center per capita is higher than in Brazil, allowing patients to be referred to specialized services closer to their place of residence, increasing survival rates and decreasing the patients’ hospitalization duration due to early admission [52], [60], [61].

Different studies have indicated that other factors that can decrease the delay time of the treatment of patients with IHD are the pre-hospital diagnosis using established protocols, and the direct referral for primary percutaneous coronary intervention in a Reference Interventional Cardiology Center, reducing the impact of the distance on the mortality rate of such patients [62], [63].

In Brazil as a whole and in Parana state, the main health care system prevalent in the country is based on a unified and universal access oriented system which directs the patients to services located in large urban centers when the case demands a more specialized attention [64].

However, as this study indicates, these specialized services are not present in all Regional Health Units. Thus, depending on the geographic location of patients suffering from IHD, which need immediate attention, these patients will be referred to a hospital closer to their primary residence that does not have a specialized service, and from there they will be transferred to a Reference Interventional Cardiology Center. This later scenario increases the distance that the patient must travel and thus delays the adequate treatment initiation.

Previous studies have indicated that the established universal policies of access to health system adopted by different countries does not necessarily lead a real universal access to all groups of the population and equally to all types of specialized services due to difficulties related with distance, availability of general practitioners, among other factors [65]–[67]. The solution to this problem could be the creation of new tertiary cardiology reference services. The establishment of an efficient network of specialized cardiology centers, however, will require trained nurses and doctors, equipment, supplies, and an adequate emergency service integrated with a pre-hospital diagnosis protocol [62], [68].

In the current healthcare scenario in Brazil, problems have been identified regarding the accessibility and equity offered by the different health care levels of assistance [8], [9], particularly concerning the care provided to patients with IHD. These problems seem to be related with a lack of adequate attention and orientation for patients with risk factors for IHD in primary health care system and a deficient provision of secondary care, the latter generating lack of access to specialized care. As a consequence, untreated patients may develop an acute heart disorder and are received in primary hospitals without the adequate structure and trained health professionals, ultimately being transferred to tertiary hospitals distant from their residence.

In 2011 the Brazilian government has created the “Emergency Care Network - Acute Myocardial Infarction Line” aiming at integrating a hierarchical and regulated network of different emergency services specifically to serve patients with ischemic heart disease [69].

The availability of an efficient Mobile Emergency Care Service is important in allowing fast dispatch, pre-hospital diagnosis, and ultimately treatment for these patients. In Brazil, a nationwide implementation initiative for an Urgency Mobile Care Service (SAMU) has been in place since 2008 [70]. The success of its implantation in most of the country represents a key element to provide proper care for patients with ischemic heart diseases. However, as our results indicate, the implementation of new Reference Interventional Cardiology Centers in Parana state is fundamental to decrease the distance between patients’ residence and the tertiary hospital. This would likely reduce the delay in treating patients with IHD, also decreasing the corresponding mortality rate secondary to IHD.

One of the possible limitations of the study was the use of secondary data, which may present under-notifications of IHD death. However, the data quality of the Mortality Information System obtained from the website of the Ministry of Health has increased its reliability [71], even so we confirmed our data completeness (number of IHD deaths cases) with the Epidemiological Surveillance System of Health Department of the State of Parana. Another limitation of the study was the fact that we do not considered in our study the existent structure of the Reference Interventional Cardiology Centers, which may be an contributing factor regarding the delay to treatment initiation. These include factors such as the number of available beds in Coronary and Chest Pain Units as well as the number of cardiologists, catheterization laboratory with a team of hemodynamicists and nurses available 24 hours/day.

Our work indicated that the number of Reference Interventional Cardiology Centers in the state of Parana, Brazil, is not sufficient. We therefore suggest that the allocation of new cardiology centers is a priority, especially in the 12 cities constituting Regional Health Units headquarters without Reference Interventional Cardiology Centers. Additionally, there is a need to improve the primary care provided by hospitals in cities where the time between services and transportation to Reference Interventional Cardiology Centers is long, specifically in cities with high rates of specific mortality by IHD.

With the growing global concern about the IHD mortality, we would like to suggest also the creation of an observatory for mapping, monitoring and prevention of IHD mortality using geospatial tools to identify the locations with the highest number of cases and deaths due to IHD, sharing information to be analyzed in the scientific context and also to formulation of local, regional and inter-regional public policy interventions.

In conclusion, our work shows that the high mortality rate by IHD within the Regional Health Units was not restricted to socioeconomic and demographic variables, but dependent on the distance of each municipality to their Reference Interventional Cardiology Center, demonstrating that geographic factors play a significant role in IHD mortality within cities.
